# Physiological effects of microcurrent and its application for maximising acute responses and chronic adaptations to exercise

**DOI:** 10.1007/s00421-022-05097-w

**Published:** 2022-11-18

**Authors:** Stefan Kolimechkov, Marcos Seijo, Ian Swaine, Jack Thirkell, Juan C. Colado, Fernando Naclerio

**Affiliations:** 1 Centre for Exercise Activity and Rehabilitation, Institute for Lifecourse Development, School of Human Sciences, University of Greenwich, London, UK; 2 Department of Biological Sciences, School of Life and Environmental Sciences, Royal Holloway University of London, London, UK; 3 Research Group in Prevention and Health in Exercise and Sport, University of Valencia, Valencia, Spain

**Keywords:** Microcurrent electrical nerve stimulation, Subsensory, Non-invasive electrical micro-ampere stimulus, Delayed-onset muscle soreness, Muscle thickness, Lipolysis, Body composition

## Abstract

Microcurrent is a non-invasive and safe electrotherapy applied through a series of sub-sensory electrical currents (less than 1 mA), which are of a similar magnitude to the currents generated endogenously by the human body. This review focuses on examining the physiological mechanisms mediating the effects of microcurrent when combined with different exercise modalities (e.g. endurance and strength) in healthy physically active individuals. The reviewed literature suggests the following candidate mechanisms could be involved in enhancing the effects of exercise when combined with microcurrent: (i) increased adenosine triphosphate resynthesis, (ii) maintenance of intercellular calcium homeostasis that in turn optimises exercise-induced structural and morphological adaptations, (iii) eliciting a hormone-like effect, which increases catecholamine secretion that in turn enhances exercise-induced lipolysis and (iv) enhanced muscle protein synthesis. In healthy individuals, despite a lack of standardisation on how microcurrent is combined with exercise (e.g. whether the microcurrent is pulsed or continuous), there is evidence concerning its effects in promoting body fat reduction, skeletal muscle remodelling and growth as well as attenuating delayed-onset muscle soreness. The greatest hindrance to understanding the combined effects of microcurrent and exercise is the variability of the implemented protocols, which adds further challenges to identifying the mechanisms, optimal patterns of current(s) and methodology of application. Future studies should standardise microcurrent protocols by accurately describing the used current [e.g. intensity (μA), frequency (Hz), application time (minutes) and treatment duration (e.g. weeks)] for specific exercise outcomes, e.g. strength and power, endurance, and gaining muscle mass or reducing body fat.

## Introduction

The human body works via bioelectricity using current in the range of pico-ampere (pA 10^−12^ A), nano-ampere (nA 10^−9^ A), and micro-ampere (μA 10^−6^ A), which are essential for all healing processes (Al-Tubaikh 2018). This was well documented by Robert Becker, ‘the father of electromedicine’ due to his investigations on salamanders (Becker and Selden 1985). In the human body, sub-sensory bio-currents influence growth, adaptation, and tissue repair, by optimising all physiological functions, including nervous system signalling, muscle growth, and remodelling (Poltawski and Watson 2009). The application of a non-invasive, externally generated sub-sensory current, known as microcurrent therapy (MCT), was developed in the 1970s. Microcurrent is referred to in the literature under several pseudonyms, including microcurrent electrical nerve stimulation (MENS), microcurrent electrical therapy, low-intensity direct current, and low-voltage micro-amperage stimulation (Belanger 2015). However, regardless of the terminologies, microcurrent describes micro-amperage current ranging from 1 to 999 μA, (i.e.  < 1 mA; 10^–3^ A) applied with frequencies between 0.5 and 100 Hz. Electrical currents of this magnitude are insufficient to excite motor nerves (Lambert et al. 2002). The application of MCT to promote health is based on the Arndt–Schulz law, which states that weak electrical stimuli increase physiological activities while strong electrical stimuli inhibit them (Al-Tubaikh 2018). Microcurrent therapy has been shown to promote increased rates of tissue synthesis, angiogenesis and neural sprouting (Poltawski et al. 2012). Furthermore, the application of microcurrent stimulates mitochondrial biogenesis by increasing adenosine triphosphate (ATP) production and lipolytic activity from both visceral and subcutaneous adipose tissue (Noites et al. 2015). For instance, in both animal and human studies, the application of MCT has been associated with several anabolic or healing processes, namely: (i) the stimulation of growth and tissue restoration (Zizic et al. 1995), (ii) diminution of oedema (Cook et al. 1994), (iii) increased myogenesis differentiation (Ohno et al. 2019), and (iv) the promotion of muscle protein synthesis (Moon et al. 2018; Ohno et al. 2019).

Although various therapeutic effects linked to the application of microcurrent have been reported, to the best of our knowledge, there is still a paucity of research describing the effects of combining MCT with physical exercise. Therefore, the purpose of this review was to summarise the physiological mechanisms mediating the effects of regular combination of MCT with different kinds of exercise modalities on cell energetics and exercise-induced adaptations. Furthermore, a more in-depth discussion on the potential mechanisms associated with MCT in healthy physically active individuals is presented.

### Differences in the way microcurrent is applied in humans

One of the most confounding variables impacting MCT outcomes is the current itself. Aside from the amplitude of the current, if it is a pulsed current, it is important to know the frequency of the pulse, how long the pulse is (i.e. pulse-width), the shape of the pulse, the direction the pulse flows in, the duration (time) of the stimulation. Some waveforms are exceedingly complex, with variations in amplitude, frequency, pulse-width, and polarity all occurring within a treatment cycle. Unfortunately, many investigations do not properly describe the characteristics of the applied current, making it difficult to elucidate what is the optimal current configuration to be applied in different circumstances and for different outcomes, e.g. maximise exercise adaptation (such as hypertrophy or body fat reduction), accelerate recovery or attenuate exercise-induced muscle damage. Furthermore, the electrode placement could also be a potential source of confusion. Nonetheless, the treatment pattern of some microcurrent devices such as the Arc4Health used by Naclerio et al. (2021) and Naclerio et al. (2019) include the delivery of ubiquitous electrical currents cycled between positive going pulses and negative going pulses (i.e. forward, and reverse polarity). Consequently, a close to an equal number of pulses will be delivered in each direction impacting the human body ubiquitously with no expected differences from the way around which the electrodes are attached.

To the best of our knowledge, no study has examined the impact of current characteristics and the effectiveness associated with the expected outcomes. The following sections review the characteristics of the used microcurrent protocols, the associated outcomes, and the probable mechanism of action.

### Application of microcurrent therapy in patients and potential benefits for healthy physically active humans

Microcurrent therapy has been used in a clinical setting for many years, and its origin dates back a few centuries ago when electrostatically charged leaf was used to treat skin conditions such as skin lesions and skin ulcers (Belanger 2015). Some of the first published peer-reviewed articles reported positive effects of MCT to treat varicose and skin ulcers in humans (Assimacopoulos 1968; Wolcott et al. 1969). Injured muscle and skin were reported to have their own ‘injury bio-currents’, which play a major role in tissue repair (Foulds and Barker 1983). Indeed, all cells in the human body generate mini-currents that facilitate cellular functioning (Vanhaesebroeck 2006). Cells in tissues can generate extracellular electrical fields which appears to control wound healing (Zhao et al. 2006) by eliciting a flow of positive charge of up to + 70 mV directed towards the wound centre (Vanhaesebroeck 2006). Microcurrent therapy has an unrealised potential in healing dysfunctional tissue because of its ability to mimic weak, natural bioelectric ‘injury currents’, and subsequently enhance wound repairing (Poltawski and Watson 2009). In addition, within the clinical setting MCT has been widely applied in patients to reduce and relieve sinus pain (Goldsobel et al. 2019; Maul et al. 2019), chronic scar pain and itch from skin scars (Perry et al. 2010; Ud-Din et al. 2013), chronic back pain (Lerner and Kirsch 1981), and partial rotator cuff tears related pain (Vrouva et al. 2019).

**Fig. 1:**
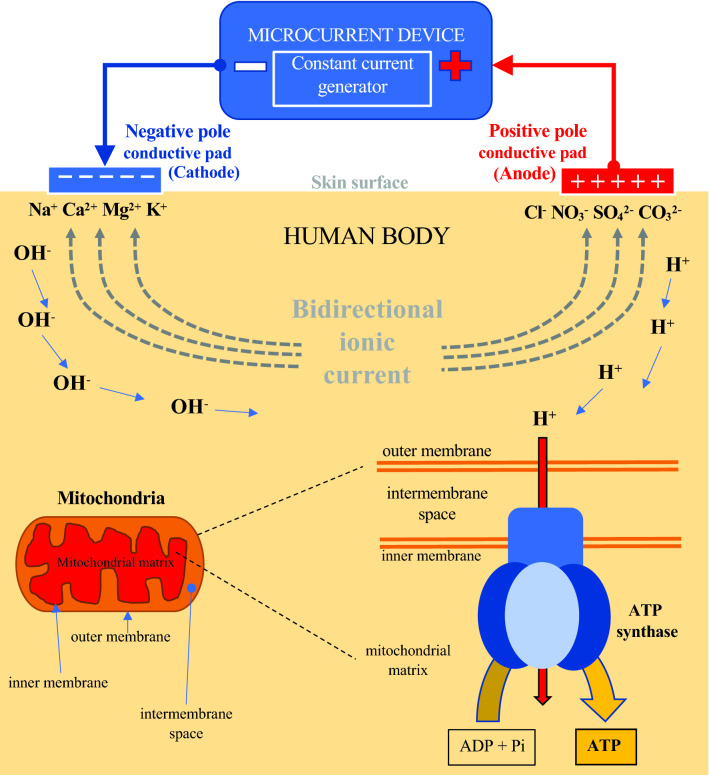
Resulted electrical under-skin current and subsequent bioelectrical (ionic) ATP formation elicited by the application of microcurrent. *ATP *Adenosine Triphosphate, *Pi *Inorganic Phosphate

As described in Fig. 1, the primary parameter of MCT is the electrical current, which in fluids such as those found in the human body, is manifest as a flow of charged particles (ions). Ions are attracted to the opposite electrical charges (negative to positive and positive to negative); hence, the cathode and anode of the electrodes placed on the human skin repel similarly charged and attract oppositely charged ions. The flow of electrons through the conducting wires occurs due to the different charges between the battery poles, thus creating a potential difference that moves the electrons (Watson and Nussbaum 2021). The direction of ion flow in the tissue is opposite to that of the electrons. This sub-sensory delivered current mimics the electrical signals found in living tissue (Poltawski and Watson 2009), and it is characterised by its frequency, shape, width, and direction of the pulse, as well as the duration of the applied stimulus.

The application of MCT on rat skin, using currents from 50 µA to 1000 µA, significantly increased ATP by between three and five-fold. The optimal level of current, eliciting the highest increase of ATP concentration, ranged between 100 and 500 µA. Indeed, ATP concentration levelled with currents exceeding 1000 µA, and decreased with currents of 5000 µA (Cheng et al. 1982). The increased ATP production can be explained by the movements of protons (H^+^). This is described by Mitchell’s chemiosmotic theory (Mitchell 1976), which states that the concentration of protons across the inner membrane of mitochondria can be used to phosphorylate ADP to ATP (ATP synthase reaction synthesises ATP through H + translocation). In the presence of a constant microcurrent, two electrodes are attached on the surface of the skin resulting in two opposite flows of ionic current (bidirectional ionic current); see Fig. 1. The positive ions move towards the negative electrode (cathode), and the negative ions move towards the positive electrode (anode). At the same time, two different electrochemical processes of decomposition of H_2_O molecules occur at the electrodes attached on the skin surface. First, two H_2_O molecules at the negative electrode (cathode) receive a total of two electrons and decompose into a molecule of hydrogen (H_2_) and hydroxyl ion (OH^−^). This is the cathodic hydrolysis processes of reduction: 2H_2_O + 2e^−^ → H_2_ + OH^−^. In the second process, two H_2_O molecules give the positive electrode (anode) a total of four electrons and decompose into a molecule of oxygen and four hydrogen ions (H^+^). This is the anode hydrolysis processes, oxidation: 2H_2_O → O_2_ + 4H^+^ + 4e^−^. Protons are formed on the anodic side, and they move from anode (+) to cathode (−). This flow of positive hydrogen ions towards the negative electrode is proposed as a mechanism of increased ATP production in the mitochondria. Molecules of ATP are formed through the H + -ATPase when they reach the mitochondrial membrane (Cheng et al. 1982).

The ability of MTC to increase ATP within the stimulated tissue in healthy physically active individuals has been shown to increase the energy available to the cells, which may facilitate muscle mass gain and maintenance throughout the lifespan and improve performance by enhancing recovery after exercise sessions in athletes. Compared to sham treatment, researchers documented that MCT applied in acute trials attenuates markers of muscle damage (Curtis et al. 2010; Kwon et al. 2017; Lambert et al. 2002), and reduces perceived exercise-induced exhaustion (Stosslein and Kuypers 2022). Indeed, two randomised controlled trial interventions confirmed the positive effect of MCT to attenuate the perception of delayed-onset muscle soreness (DOMS, a marker of muscle damage) in both resistance-trained (Naclerio et al. 2019) and endurance athletes (Naclerio et al. 2021). Another proposed benefit of MCT for healthy active individuals is its effect to reduce body fat. A significant reduction of visceral and subcutaneous abdominal fat was observed in university female students after 5 weeks of MCT added to 30 min of aerobic exercise (Noites et al. 2015). Similarly, our study with endurance athletes suggested that the implementation of post-exercise MCT for 3 h over 8-week period decreased lower limb fat compared to exercise alone (Naclerio et al. 2021).

### Effects of microcurrent in enhancing the effects of exercise

#### General background

Previous reviews (Balakatounis and Angoules 2008; Iijima and Takahashi 2021; Lambert and Burgess 2004; Mercola and Kirsch 1995; Poltawski and Watson 2009; Yu et al. 2014) focussed on the effects of MCT to alleviate pain and promote tissue healing. To the best of our knowledge, there is a paucity of research concerning the mechanistic effects of combining microcurrent with exercise to optimise its effects in healthy, non-injured individuals. Combining MCT with exercise has been hypothesised to improve exercise performance, recovery, and morphological or structural changes in skeletal muscles (Kwon et al. 2017; Naclerio et al. 2019). The proposed mechanisms associated with MCT are: (i) replenishment of ATP (Cheng et al. 1982), (ii) maintenance of intercellular calcium homeostasis which in turn optimises exercise-induced structural and morphological adaptations (Lambert et al. 2002), and (iii) eliciting a hormone-like effect that increases catecholamine secretion (Al-Tubaikh 2018). Table 1 summarises the main characteristics, the study sample size, and documented effects of applied MCT protocols in human studies.

**Table 1:** Effects of MCT on exercise adaptation in humans

Study	Description of study	Design	Microcurrent parameters	Placement of electrodes	Population	Effects
(Stosslein and Kuypers 2022)	Controlled study (two-way crossover study, *n* = 20)	Acute trial: 30 min MCT post-exercise (resistance training)	200 μA with frequency of 0.3–3.5 Hz frequency-specific microcurrent	4 anatomical points: upper buttock, lower buttock, lower back, upper neck	18–40 year-old males with resistance training experience	↑ self-ratings of well-rested, sociable. ↓ Feeling of exercise-induced exhaustion
(Vilarinho et al. 2022)	Controlled study (MCT: *n* = 18; Sham: n = 20)	Acute trial: 40 min MCT pre-exercise followed by 50 min moderate aerobic exercise on a cyclo-ergometer	1000 μA with frequency of 25 Hz during the first 20 min followed by 10 Hz during the last 20 min	Abdominal region	Mean age of 20.6 ± 1.8 years men and women (university students)	No difference in lipolysis
(Naclerio et al. 2021)	Controlled study (MCT: *n* = 9; Sham: *n* = 9)	Long-term trial: 3 h/day MCT + endurance training × 8 weeks	50–400 μA with frequency of ∼1000 Hz	Dominant leg	18–45-year-old male cross-country athletes	Positive changes in body composition:↓ leg fat with a trend towards ↓whole-body fat → body mass ↓DOMS
(Piras et al. 2021)	Controlled study (*n* = 10)	Acute trial: 20 min MCT pre-exercise and 20 min post-exercise	400 µA with frequency of 256 Hz rectangular waveform (1 s of impulse duration)	Right leg (quadriceps) using transducer gloves to massage the quadriceps	27.2 ± 3.6-year-old recreationally active healthy men and women	Faster recovery after cycling exercise
(Naclerio et al. 2019)	Controlled study (MCT: *n* = 9; Sham: *n* = 9)	Long-term trial: 3 h/day MCT + resistance training × 8 weeks	50–400 μA with frequency of ∼1000 Hz	Dominant leg	18–45-year-old Trained men	Maximised muscular architectural changes ↓ DOMS
(Kwon et al. 2017)	Controlled study (MCT: *n* = 19; Sham: *n* = 19)	Acute trial: 40 min MCT with muscle function tests before and after	25 μA with frequency of 8 Hz alternating current with monophasic rectangular pulse format	8 anatomical points of the dominant arm and leg	65 years of age and above elderly men and women	Enhanced some muscle function: ↑ handgrip strength
(Noites et al. 2017)	Controlled study (MCT: *n* = 42; Sham: *n* = 41)	Acute trial: 40 min MCT + 60 min aerobic session with moderate-intensity	700–999 μA with a frequency of 25 Hz for the first 20 min, and 10 Hz for the last 20 min bipolar square-wave, alternated with electrical current using transcutaneous band electrodes	Lower abdominal region	18–30-year-old men and women (university students)	Induces lipolysis
(Noites et al. 2015)	Controlled study (MCT-1: *n* = 9; MCT-2: *n* = 9; MCT-3: *n* = 7; MCT-4: *n* = 8; Sham: *n* = 9)	Long-term trial: 30 min MCT + 30 min aerobic moderate-intensity exercise (twice a week for 5 weeks, total of 10 sessions)	1–999 μA with frequency of 25–10 Hz and 25–50 Hz (monophasic and rectangular microcurrent with polarity changes every second)	Abdominal region	18–30-year-old women (university students)	Induces lipolysis and provides effects additive to aerobic exercise on fat tissue decrease
(Curtis et al. 2010)	Controlled study *n* = 35 (MCT on 1 leg; Sham on the other)	Acute trial: 20 min MCT (frequency-specific microcurrent) after eccentric muscle contractions on a seated leg machine	200 μA with different frequencies on channel A and B	Leg	20–40 year-old healthy and recreationally active men and women	↓ DOMS
(Lambert et al. 2002)	Controlled study (MCT: *n* = 15; Sham: *n* = 15)	Acute trial: 96 h MCT (electro-membrane microcurrent therapy) after eccentric contractions of the nondominant elbow flexor muscles	20 μA	Exercised upper-arm	29.5 years mean age healthy men	↓ DOMS
(Allen et al. 1999)	Controlled study (MCT: *n* = 9; Sham: *n* = 9)	Acute trial: 20 min MCT 24, 48, and 72 h after eccentric contractions of the nondominant arm (total of 60 min MCT)	200 μA with frequency of 30 Hz for 10 min, followed by 100 μA with frequency of 0.3 Hz for 10 min	Exercised upper-arm	20.3 years mean age healthy men (*n* = 3) and women (*n* = 15)	No beneficial effect for DOMS

↑ increased,→ maintained, ↓ decreased

#### Effect of microcurrent on body fat and exercise-induced lipolysis

Only a few numbers of studies analysed the impact of combining MCT with exercise interventions on body fat and exercise-induced lipolysis. A significant reduction of visceral and subcutaneous abdominal fat was observed in young female university students (18–30 years of age) when using MCT during or shortly after the completion of a 30 min endurance exercise vs performing the exercise alone (Noites et al. 2015). The MCT was locally applied (twice a week for 5 weeks, total of 10 sessions) on the abdominal region with currents ranging between 1 and 999 μA, delivered between 25 and 50 Hz of frequency. The percentage of reduction in visceral and subcutaneous abdominal fat for the three groups (*n* = 8–9) using transcutaneously applied MCT was ~ 12–20%, 12–12% and ~ 10–13%, respectively. Conversely, smaller reductions of both visceral (~ 7%) and abdominal (~ 4%) fat were observed in the group performing only exercise (*n* = 9). Similarly, our research group analysed the effect of using MCT for 3 h post-workout or during the morning on non-training days for 8 weeks in 18 cross-country male athletes (Naclerio et al. 2021). The microcurrent protocol used an intensity range of 50–400 μA with a fundamental frequency of ∼1000 Hz. Compared to the non-MCT treatment (only training, *n* = 9), the athletes allocated to the MCT group (*n* = 9) significantly (*p* < 0.05) decreased lower limb fat and showed a trend (*p* > 0.05 and < 0.1) to reduce whole-body fat (Naclerio et al. 2021).

Noites et al. (2017) investigated the acute effect of combined MCT with endurance exercise on normal and overweight (body mass index between 18.5 and 29.9 kg/m^2^) male and female university students aged 18–30 years. Participants received a trans-abdominal microcurrent stimulation (MCT group, *n* = 42) or placebo (exercise alone group, *n* = 41) for 40 min prior to performing a single moderate aerobic exercise session. The MCT protocol had an intensity between 700 and 999 μA with a frequency of 10–25 Hz. Although both groups (MCT and placebo) showed significant increases (*p* < 0.01) and large effect sizes (*d* = 2.75 and 2.5 respectively) in the exercise-induced serum glycerol levels that were used as lipolysis marker indicating triglycerides hydrolysis (Lafontan and Langin 2009), the elevated values measured in the MCT group were meaningful compared to those measured in the placebo group (+ 0.15 ± 0.09 vs. 0.09 ± 0.07 mmol/l, *p* = 0.03). However, more recently, compared to a sham treatment (*n* = 20), no lipolytic effect was observed in response to a 40 min application of MCT ($\le 1000$ μA at 10 and 25 Hz) prior to 50 min of moderate-intensity endurance exercise (*n* = 18) in university students (Vilarinho et al. 2022). Further studies, using larger sample sizes, similar to those used by Noites et al. (2017) (i.e. *n* = ~ 40 per treatment), comparing the effectiveness of different MCT protocols administered with different currents and frequencies are needed to establish the lipolytic effect of MCT, as well as the optimal time of application (e.g. before, during, after exercise time). Moreover, in order to explore the lipolytic effect of MCT, either delivered alone or combined with exercise, investigations using individuals with different adiposity levels (i.e. overweight and obese participants) are needed.

**Fig. 2:**
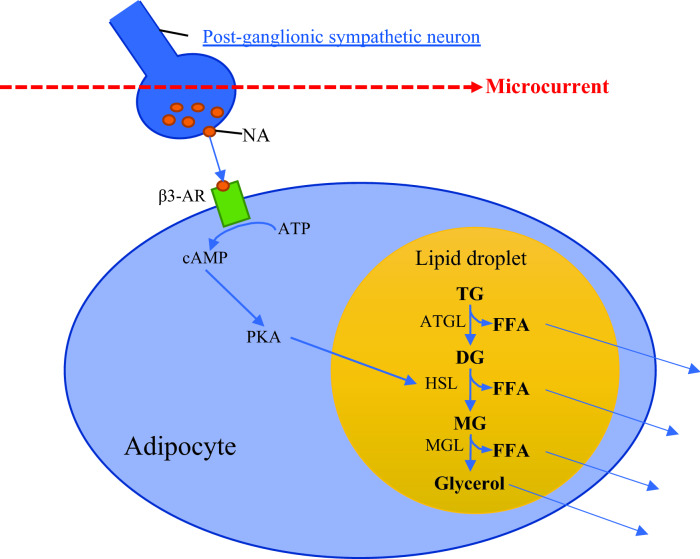
Schematic representation of the microcurrent-induced lipolysis. *NA* noradrenaline, *β3-AR* beta3 adrenoreceptor, *ATP* adenosine triphosphate, *cAMP* cyclic adenosine monophosphate, *PKA* protein kinase A, TG, DG and, MG tri-, di, and monoglycerides, ATGL, HSL, and MGL lipolytic enzymes, *FFA* free fatty acids

The chronic effect of MCT to increase the exercise-induced lipolytic activity in healthy individuals (Noites et al. 2017, 2015) can be explained by the voltage-dependent potassium currents of the human adipose cells (Ramirez-Ponce et al. 2003), which makes lipolysis more sensitive to MCT. For instance, combined mild electrical stimulation (1.4 ± 0.1 V/cm) and heat shock induced by electro-conductive and thermo-generative rubber electrodes reduced visceral adiposity in metabolic syndrome and type 2 diabetes patients (Kondo et al. 2014). Even higher currents (4 mA) have been demonstrated to significantly increase lipolysis in humans, and this effect could be explained by the activation of cyclic adenosine monophosphate (cAMP), which subsequently stimulates lipolysis (Hamida et al. 2011). Although the exact mechanism by which MCT can reduce local adiposity has not been fully understood, it could be explained by the MCT stimulatory effect on the sympathetic nervous system (SNS). The primary purpose of the SNS is to stimulate the body’s fight or flight response, while remaining constantly active to maintain homeostasis (Motiejunaite et al. 2021). Microcurrent therapy has been suggested to exert ‘hormonelike effects’ caused by increased catecholamine secretion (noradrenaline) from the postganglionic sympathetic neuron of the nervous system and cell membrane G protein (Al-Tubaikh 2018). The postganglionic neurons extend across most of the human body, and when the currents react with them, the secretion of noradrenaline increases, binding onto β3-adrenoreceptor (β3-AR), which in turn converts ATP into cAMP in adipocytes (Noites et al. 2017), see Fig. 2. The increased concentration of cAMP activates the enzyme protein kinase A (PKA), which promotes lipolysis by stimulating the reactions catalysed by the adipose triglyceride lipase (ATGL), hormone-sensitive lipase (HSL), and monoglyceride lipase (MGL). Briefly, as described in Fig. 2, triglycerides are then broken down inside the lipid droplet into one free fatty acid and diglyceride by the ATGL enzyme. Then, diglyceride is metabolised to one free fatty acid and monoglyceride by the HSL enzyme, and the remaining monoglyceride is eventually metabolised to one free fatty acid and glycerol molecule by MGL enzyme. Thus, MCT appears to induce lipolysis throughout the stimulation of the postganglionic sympathetic neuron (Fig. 2). The glycerol and free fatty acids molecules are then released out of the adipocyte into the blood stream.

Overall, there is some evidence suggesting that MCT combined with endurance exercise can further stimulate exercise-induced lipolysis. However, the limited available evidence precludes us from making conclusive recommendations on the optimal effective lipolytic protocols. Nonetheless, MCT with intensities of 50 to 999 μA and frequencies between 10 and 1000 Hz have demonstrated to be beneficial for activating lipolysis.

#### Effect of microcurrent on skeletal muscle remodelling, structure, and size

Several animal-based studies explored MCT effects on skeletal muscles (Fujiya et al. 2015; Moon et al. 2018; Ohno et al. 2019, 2013; Park et al. 2018). In vitro, MCT with an intensity of 10 μA applied for 15–60 min and 20 μA for 15–30 min significantly increased myotubes protein content compared to untreated control level. In addition, MCT upregulated creatine kinase MM isoform (marker of myogenic differentiation), showing signs of improved membrane repair in previously injured skeletal muscle (Ohno et al. 2019). These results support the application of MCT as a potential treatment for injury rehabilitation. MCT was hypothesised to induce intracellular signals involved in protein synthesis of atrophied and/or injured skeletal muscles. A 60 min MCT protocol with an intensity of 10 μA and frequency of 0.3 Hz conducted on three non-consecutive days facilitated regrowth of atrophied soleus muscle in mice (Ohno et al. 2013). Similarly, an identical MCT protocol implemented three times a week for three weeks facilitated the regeneration of injured tibialis anterior muscle in mice (Fujiya et al. 2015). These aforementioned studies strongly suggested a potential stimulation of MCT on muscle satellite cells proliferation. Indeed, two subsequent studies conducted with rabbits (Moon et al. 2018; Park et al. 2018), using a similar MCT protocol (applied for 60 min daily for 2 weeks) demonstrated a greater regenerative effect in atrophied gastrocnemius muscle. The MCT protocol with lower intensity (20 μA) promoted greater regenerative effect of atrophied rabbit muscle in comparison with high-intensity (5000 μA) or sham (Park et al. 2018).

In patients with first-time anterior cruciate ligament rupture, MCT reduced skeletal muscle fibre atrophy of the quadriceps (Toth et al. 2020). It suggests that MCT optimises cellular regeneration and consequently attenuates impairment of both muscle fibre size and contractility which is markedly reduced three weeks post-surgery. In line with previous veterinary studies (Fujiya et al. 2015; Ohno et al. 2019, 2013; Park et al. 2018), the aforementioned intervention in humans supports the benefits of MCT to accelerate post-surgery healing and attenuate muscle atrophy in humans.

To the best of our knowledge, only one study conducted in our laboratory has analysed the effect of adding MTC to resistance training in healthy young recreationally trained men (Naclerio et al. 2019). A potential additive effect of MCT to optimising hypertrophy (enlargement of muscle thickness and pennation angle) was observed in the vastus medialis, vastus lateralis, and brachialis muscles in young resistance-trained men after 8 weeks of MCT (3 h a day with an intensity of 50 and 400 μA, and a frequency of ~ 1000 Hz) post-workout or in the morning of non-training days (Naclerio et al. 2019). The enlargement of muscle thickness along with a greater pennation angle of muscular fibres provides a larger fibre area for a given volume of muscle and therefore, more potentially activated actin-myosin cross bridges, resulting in greater strength and force generation (Suetta et al. 2008). Accordingly, it seems that post-workout MCT can create summative microcurrent-induced hypertrophic effects by optimising muscle thickness enlargement and greater pennation angles. Nonetheless, it is worth highlighting that the observed differences between the two interventions groups, did not reach statistical significance. Although training is considered the most effective stimulus to obtain exercise-related outcomes (e.g. hypertrophy, muscular function enhancement, etc.) (Bischoff-Ferrari and Dawson-Hughes 2019), the length of the training programme, i.e. 8 weeks, in well-trained participants can be considered enough to elicit the observed training adaption. Nevertheless, such training length could still be insufficient to elicit statistically significant differences produced by the application of MCT compared to the adaptations obtained by the training intervention alone.

**Fig. 3:**
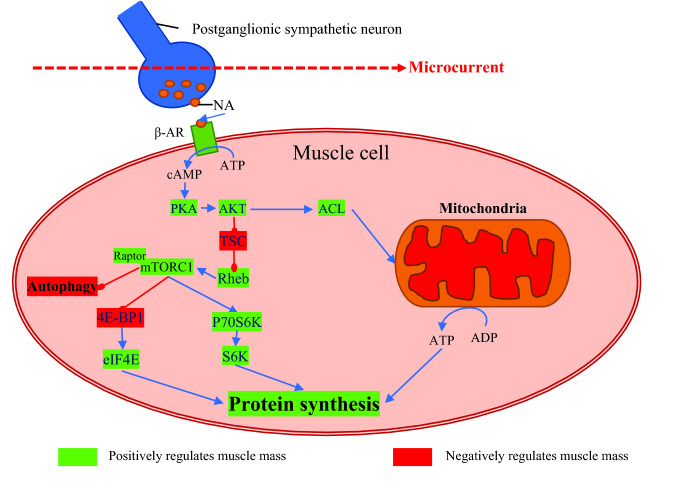
Muscle protein synthesis activation by the application of microcurrent. *NA* noradrenaline, *β3-AR* beta3 adrenoreceptor, *ATP* adenosine triphosphate, *cAMP* cyclic adenosine monophosphate, *PKA* protein kinase A, *AKT* Ak strain transforming/protein kinase B, *ACL* ATP citrate lyase, *ADP* adenosine diphosphate, *TSC* tuberous sclerosis, *Rheb* Ras homolog enriched in brain, *mRORC1* mammalian target of rapamycin complex 1 characterised by the presence of Raptor, *4E-BP1* factor 4E-binding protein 1, *eIF4E* eukaryotic translation initiator factor 4E, *P70S6K* Ribosomal protein S6 kinase beta-1

The mechanistic effect of MCT on skeletal muscles could be explained by its stimulatory effect on noradrenaline secretion, which has been proposed as an additional mechanism for promoting muscle protein synthesis (Navegantes et al. 2002). Catecholamine secretion was proposed to be increased following MCT application (Noites et al. 2017). For instance, increased noradrenaline secretion has been recently reported in mice after transcranial direct current stimulation (Mishima et al. 2019). Noradrenaline is usually associated with the stimulation of lipolysis (see Fig. 2) and glycogenolysis through an increase in intracellular cAMP. Considerable evidence also suggests that catecholamines may have an anabolic effect on skeletal muscle protein metabolism (Navegantes et al. 2002). In addition, noradrenaline may increase the rate of protein synthesis in oxidative muscles, which lead to increased protein accretion (Navegantes et al. 2002). We hypothesised that the increased noradrenaline secretion by MCT enhances muscle protein synthesis using similar pathways as those of clenbuterol. Previous reviews reported that oral administration of clenbuterol induces hypertrophy of skeletal muscle in different species (Mersmann 1998). Indeed, clenbuterol was well documented to be one of the main pathways involved in protein synthesis, which promotes hypertrophy by the activation of mammalian target of rapamycin complex 1 (mTORC1) (Vainshtein and Sandri 2020). Similarly, we assume that the potential MCT-induced secretion of noradrenaline would trigger intracellular pathways contributing to further protein synthesis stimulation (Fig. 3).

#### Effect of microcurrent on exercise-induced delayed-onset muscle soreness (DOMS)

Delayed-onset muscle soreness is associated with muscular discomfort and stiffness in muscles after performing unfamiliar physical activity (Cleak and Eston 1992). DOMS is described as disrupted and sore muscle tissue in addition to an inflammatory response (Connolly et al. 2003). DOMS has been attributed to disturbance of calcium homeostasis, muscle fibre disruption, and firing of IV nerves in response to products of macrophage activity and intracellular contents accumulated in the interstitium (Armstrong 1984; Radak et al. 2012). However, recent research suggests a strong involvement of the connective tissue (Wilke and Behringer 2021). Particularly the deep fascia would be intimately linked with the underlying skeletal muscle and may therefore be damaged (suffering micro-ruptures and inflammation) during unaccustomed hard exercises bouts and thereafter elicit perceived post-exercise discomforts (Wilke and Behringer, 2021).

Although different treatments have been proposed to attenuate the severity of DOMS, their efficacy remains inconsistent with both positive and negative results (Connolly et al. 2003). Studies have demonstrated that MCT can reduce the severity of symptoms of exercise-induced muscle damage in variety of populations after the completion of both acute and long-term exercise protocols (Table 1). For instance, electro-membrane microcurrent therapy applied for a period for 96 h reduced the severity of DOMS symptoms in healthy men in a double-blind, placebo-controlled trial after performing eccentric contractions of the nondominant elbow flexor muscles (Lambert et al. 2002). Similarly, the application of MCT using a specific frequency (a modality of MCT including a particular list of frequencies designated to target specific tissues or health-related conditions) for 20 min attenuated the perception of DOMS in the MCT treated leg vs sham treated leg (Curtis et al. 2010). In addition, a similar specific MCT applied for 30 min post-exercise in healthy resistance-trained young men (18–40 years old) reduced exercise-induced exhaustion based on self-reported questionnaires (Stosslein and Kuypers 2022). Furthermore, compared to a sham treatment MCT significantly decreased the symptoms of DOMS over 72 h post-exercise in cross-country male athletes (Naclerio et al. 2021) and at 12, 24, and 48 h after performing an exercise-induced muscle soreness protocol in resistance-trained men (Naclerio et al. 2019). Nevertheless, a study using only two distinct currents (100 and 200 μA), with frequencies set at 0.3 and 30 Hz, and using a shorter application time of up to 20 min (Allen et al. 1999), reported no reduction in DOMS.

The mechanisms by which MCT attenuate the severity of DOMS are still unclear. It has been proposed that acute or regular MCT application attenuate disturbances of the intracellular calcium homeostasis (Lambert et al. 2002), which result from exercise-induced muscle damage protocols mainly those including a high degree of eccentric muscular activation (Armstrong 1984; Radak et al. 2012).

Overall, the available evidence supports the beneficial effect of MCT to attenuate the perception of DOMS in physically active individuals. Effective MCT protocols involve complex stimulation patterns, which simultaneously deliver currents of different strengths (e.g. 20–400 μA) and long applications times of up to 3 h.

#### Effects of microcurrent when combined with endurance exercise

There have been a few acute (Noites et al. 2017; Piras et al. 2021; Vilarinho et al. 2022) and long-term (Naclerio et al. 2021; Noites et al. 2015) studies combining MCT with endurance exercises. Compared to a sham treatment group (*n* = 41), adding MCT (*n* = 42) to a 60 min aerobic session acutely increased lipolysis in young (18–30 years old) men and women. The microcurrent protocol was set at an intensity between 700 and 999 μA with frequencies of 10–25 Hz (Noites et al. 2017). Conversely, a recent study reported no acute lipolytic effect of a pre-exercise MCT protocol set at 1000 μA intensity in young university students (Vilarinho et al. 2022). On the other hand, MCT was implemented in young physically active individuals (men and women aged 27.2 ± 3.6 years) before and after endurance exercise (Piras et al. 2021). The authors suggested that MCT prior to heavy endurance cycling exercises improved recovery by increasing oxygen extraction at muscle microvasculature. Furthermore, compared to the control condition, post-exercise MCT improved recovery through faster parasympathetic reactivation. The MCT involved a pre- and post-exercise 20 min protocol set at 400 µA and 256 Hz (Piras et al. 2021).

Despite some controversy observed in the aforementioned acute studies, it is suggestive that combining MCT with endurance training can eventually maximise exercise-induced lipolysis. For instance, MCT using currents from 1 to 999 μA, combined with moderate-intensity endurance training (twice a week for 5 weeks) significantly decreased subcutaneous fat measured by ultrasonography in young females participants (18–30 years old), compared to performing exercise alone (Noites et al. 2015). The observed effects were maintained for 4 weeks after the completion of the study. The authors reported that lower frequencies protocols (10–25 Hz) are more favourable to stimulate visceral fat reduction than higher (25–50 Hz) frequencies (Noites et al. 2015). Nonetheless, it is worth emphasising the significant decrease in lower body limb fat mass observed in male cross-country athletes who wore a microcurrent device for 3 h post-workout (Naclerio et al. 2021). The MCT protocol involved lower intensities (50–400 μA) and higher frequencies (∼1000 Hz) than those applied in aforementioned trial (Noites et al. 2015). Moreover, the athletes using MCT maintained their body mass and showed a trend towards decreasing whole-body fat compared to those allocated in the sham (control) training group (Naclerio et al. 2021).

The available evidence suggests that MCT, combined with endurance exercises, may further stimulate fat metabolism, and accelerate post-exercise recovery. Such benefits are apparently associated with MCT sessions using low (50–400 μA) and high (1 to 999 μA) currents set at various frequencies. Nevertheless, protocols with lower frequencies (10–25 Hz) seem more effective in maximising exercise-induced lipolysis.

#### Effects of microcurrent when combined with resistance and power exercise

To the best of our knowledge, two acute (Kwon et al. 2017; Stosslein and Kuypers 2022) and one interventional (Naclerio et al. 2019) randomised trials have analysed the effect of MCT to maximise muscle function adaptation resulting from resistance exercise. A significant acute increase in handgrip strength (pre 26.2 ± 8.8 to post 27.1 ± 8.7 kg, *p* = 0.014) and muscular efficiency estimated by a markedly reduced root mean square values of the electromyography signals, along with an increased number of plantar flexions (pre 28.7 ± 9.5 to post 34.3 ± 6.4 repetitions, *p* = 0.006) performed in a single leg heel-rise test was reported in elderly individuals (*n* = 19) after receiving a 40 min MCT with an intensity of 25 μA and a frequency of 8 Hz (Kwon et al. 2017). Similarly, meaningful improvements in self-rated recovery and mood were observed in resistance-trained individuals (18–40 years old, *n* = 20) after receiving a 30-min frequency-specific microcurrent post-exercise with an intensity of 200 μA and frequency of 0.3–3.5 Hz (Stosslein and Kuypers 2022). These beneficial effects of MCT have been explained by an enhanced intracellular Ca^2+^ homeostasis and Ca^2+^-dependent excitation–contraction coupling that might be impaired after performing exhaustive exercise bouts (Kwon et al. 2017).

The only interventional study combining MCT and resistance training was conducted by our research group (Naclerio et al. 2019). Our results suggest that a 3 h post-workout MCT treatment with intensities set between 50 and 400 μA and ∼1000 Hz frequency may optimise some of the investigated performance outcomes (vertical jump and upper body strength). Although no difference between groups (MCT *n* = 9 vs. Sham *n* = 9) was observed for vertical jump and strength performance, only the MCT group significantly improved vertical jump height, while both groups, MCT and sham (only training treatment) similarly improved bench press strength. It was suggested that differences in the training routine configuration, involving a higher volume of work on the lower body than on the upper body musculature could have caused the observed difference (Naclerio et al. 2019). Although speculative, we hypothesise that MCT can provide advantages by maximising resistance training outcomes (e.g. strength and hypertrophy).

The limited available evidence regarding the effect of combining MCT with strength and power exercise programmes precludes us from drawing definitive conclusions and recommendations. More interventional studies using larger sample sizes [*n* > 15 per group as suggested by the results observed in the study by Naclerio et al. (2019)], including athlete and non-athlete individuals of different ages and sports disciplines, are necessary. Nonetheless, based on the available literature, it is possible to suggest that combining resistance training with MCT using intensities of 25–400 μA may optimise muscle function adaptation and exercise performance outcomes.

## Side effects of using microcurrent in healthy humans

MCT is a safe modality with very few documented side effects. A recent meta-analysis on the use of MCT to reduce musculoskeletal pain concluded that there is no severe adverse effect of microcurrent application in humans (Iijima and Takahashi 2021). The authors summarised adverse events from a total of nine trials which collectively included 281 patients. It was reported that there was only one dropout (0.4%) due to a perceived tingling in the feet after the first treatment session. Furthermore, no serious adverse events requiring medical treatment were reported. In a trial examining the safety and efficacy of MCT for sinus pain and congestion, it was concluded that MCT is safe with only minor side effects (tingling in the feet), which were resolved without medical intervention (Goldsobel et al. 2019). MCT has been applied in many medical scenarios, being reported as a safe therapy over the decades (Al-Tubaikh 2018; Xu et al. 2021). Indeed, in the two interventional studies conducted by our research group, no side effects associated with the use of MCT were reported (Naclerio et al. 2021, 2019). Similarly, no side effects were reported after acute and long-term interventions combining MCT with physical exercise in general populations (Noites et al. 2017, 2015) and in the elderly (Kwon et al. 2017). Therefore, based on the documented evidence, MCT appears to be a safe treatment modality for healthy humans, including athletes and the elderly.

## Future directions—new aspects to be considered for the application of combined microcurrent and exercise in humans

### General background

MCT combined with exercise deserves particular attention in future studies focussed on improving health and well-being. The likely beneficial effects of combining MCT with exercise interventions to maximise the exercise-induced benefits on muscular function (Kwon et al. 2017) and body composition (Naclerio et al. 2021; Noites et al. 2017, 2015) has opened the avenue for future investigations in the area of anti-ageing. There is a need to standardise the configuration of MCT protocols that focus on different exercise outcomes: (i) strength and power performance, (ii) endurance performance, (iii) muscle mass increase (hypertrophy), (iv) body fat reduction, (v) acceleration of post-workout recovery. Researchers are encouraged to accurately describe the implemented MCT protocols, including the characteristics of the current: intensity (μA), frequency (Hz), and duration of each singular application (minutes) and the entire treatment (e.g. weeks) used to maximise the aforementioned expected outcomes.

### Can microcurrent support health and wellbeing in humans?

MCT has the potential to reduce the severity of post-exercise DOMS (Curtis et al. 2010), as well as to improve exercise-induced adaptations in humans, e.g. enhance muscle function, preserve or increase lean mass, etc. (Kwon et al. 2017), reducing fat (Noites et al. 2015) or enhance recovery from exercise (Piras et al. 2021). Moreover, there is evidence that MCT provides multiple benefits to, improve healing capacity after injuries by 200%, reducing blood concentration of inflammatory cytokines (Al-Tubaikh 2018), attenuating shoulder and knee pain (Iijima and Takahashi 2021), and accelerating wound healing (Balakatounis and Angoules, 2008).

### Can microcurrent counteract the ageing process when implemented alone or combined with physical training?

Resistance training combined with a healthy diet is considered the hallmark of prevention and treatment of the age-related decline in muscle mass and muscle function (Bischoff-Ferrari and Dawson-Hughes 2019). MCT has been reported as a practical and effective method to maximise exercise-induced benefits in healthy physically active individuals (Curtis et al. 2010; Lambert et al. 2002; Noites et al. 2017, 2015; Piras et al. 2021). However, to the best of the author’s knowledge, no formal research has been conducted to verify the effects of MCT added to resistance exercises on muscle strength and physical function in non-trained middle-aged and older adults. Therefore, it is important to determine to what extent the application of MCT added to physical exercises can attenuate the age-related decline of muscular function, help to prevent sarcopenia, and the overall ageing process. Attenuating the age-related loss in muscle mass and strength is essential to preserve independent living and quality of life with ageing. The loss of muscle mass with ageing was first reported nearly a century ago by Critchley (Critchley 1931) and then named ‘sarcopenia’ by Rosenberg in 1989 (Rosenberg 1989). Nowadays, the prevalence of sarcopenia is growing worldwide. The estimated sarcopenia rates in the elderly in Europe are expected to increase nearly 64% from 19.7 million in 2016 to 32.3 million in 2045 (Ethgen et al. 2017). Considering the previously described potential benefits of MCT in attenuating muscle mass loss and slowing the progressive age-related decrease in functional capacity, there is justification for further study of the possible effect of MCT in improving wellbeing and health in older people, especially in those, particularly at risk of sarcopenia and physical frailty. However, peer-reviewed research on the effects of MCT in middle-aged and older adults are scarce. A randomised, double-blinded, sham-controlled clinical trial suggested that microcurrent may enhance physical activities and support health and wellbeing in elderly participants (aged 65 years and above) by improving functional capacity (Kwon et al. 2017). Future research should build upon those findings and determine whether MCT either alone or in combination with different exercise modalities will attenuate the age-related decline in muscle mass and improve functional capacity and wellbeing in the older population.

## Summary and conclusions

The main mechanisms of actions elicited by combining MCT with exercise in physically active individuals can be summarised as follows: (i) increased ATP resynthesis, (ii) maintenance of intracellular calcium homeostasis, (iii) enhanced exercise-induced lipolysis and (iv) promoted muscle protein synthesis.

Within an exercise training context, MCT can accelerate post-exercise recovery, attenuate the severity of delayed-onset muscle soreness, and optimise physical training outcomes, e.g. skeletal muscle hypertrophic response, strength gains, endurance capacity and fat mass reduction.

The available literature suggests that the abovementioned effects can be achieved when MCT protocols are applied incorporating currents set between 20 and 400 μA, in addition to longer application times (> 30 min up to 3 h). Nonetheless, the variability of the analysed MCT protocols adds further challenges in identifying optimal protocols in terms of the pattern of current(s), time of application and treatment duration associated with the expected specific clinical or exercise-related outcomes.

## Practical application and future perspectives

From a practical perspective, MCT can be applied for 30–40 min prior to and after endurance exercises sessions to acutely stimulate lipolysis and accelerate tissue recovery. MCT can be regularly implemented during 3 h post-workout and at some point (e.g. morning time) on non-training days to maximise training-induced adaptations and attenuate DOMS after performing unfamiliar hard physical exercises. Even though no conclusive recommendation can be made regarding the optimal current characteristics, clinicians and coaches should consider current intensities set between 20 to 400 μA to attenuate DOMS or 20–999 μA with low frequencies (10–25 Hz) to maximise lipolysis and fat reduction.

Based on the current reviewed literature, the following considerations are proposed for futures studies:

(i) Well-designed interventions, including participants of different ages (including older adults), sport disciplines, and level of performance should be designed to explore the optimal effective protocols associated with differentiated exercise-related outcomes, such as gaining muscle mass, reducing body fat, and improving recovery, strength, endurance, or power performance, and to attenuate the age-related decline in muscle mass and improving wellbeing.
(ii) In order to identify the real impact of using MCT alone or combined with exercise, researchers are encouraged to conduct a thorough control of the training load configuration in terms of intensity, rest periods, volume, training frequency, duration and exercise modalities.
(iii) The impact of some common uncontrolled variables, such as sleeping time and quality, mental wellbeing, diet patterns, and nutritional habits, should also be considered. For instance, combined approaches including the application of MCT alongside the ingestion of nutritional supplements such as high-quality protein or β-hydroxy-β- methylbutyrate (HMB), which is nitrogen-free and recommended for the elderly to counteract sarcopenia (He et al., 2016), deserves special consideration for researchers and clinicians looking effective strategies to promote health in older adults.

## Abbreviations

ATP: Adenosine triphosphate
β3-AR: β3 Adrenoreceptor
cAMP: Cyclic adenosine monophosphate
DOMS: Delayed-onset muscle soreness
HSL: Hormone-sensitive lipase
Hz: Hertz (one cycle per second)
μA: Microampere (10^−6^ amperes)
mTORC1: Mammalian target of rapamycin complex 1
MCT: Microcurrent therapy
MENS: Microcurrent electrical nerve stimulation
mA: Milliampere
MGL: Monoglyceride lipase
PKA: Protein kinase A
SNS: Sympathetic nervous system
V: Volt
